# HHV-8 encoded LANA-1 alters the higher organization of the cell nucleus

**DOI:** 10.1186/1476-4598-6-28

**Published:** 2007-04-13

**Authors:** György Stuber, Karin Mattsson, Emilie Flaberg, Emrah Kati, Laszlo Markasz, Julie A Sheldon, George Klein, Thomas F Schulz, Laszlo Szekely

**Affiliations:** 1 Department of Microbiology, Tumor and Cell Biology (MTC) and Center for Integrative Recognition in the Immune System (IRIS), Karolinska Institute, Stockholm, Sweden; 2 Department of Virology, Hannover Medical School, Hannover, Germany; 3 Department of Pediatrics, University of Debrecen, Medical and Health Science Center, Debrecen, Hungary

## Abstract

The latency-associated nuclear antigen (LANA-1) of Human Herpes Virus 8 (HHV-8), alternatively called Kaposi Sarcoma Herpes Virus (KSHV) is constitutively expressed in all HHV-8 infected cells. LANA-1 accumulates in well-defined foci that co-localize with the viral episomes. We have previously shown that these foci are tightly associated with the borders of heterochromatin [1]. We have also shown that exogenously expressed LANA-1 causes an extensive re-organization of Hoechst 33248 DNA staining patterns of the nuclei in non-HHV-8 infected cells [2]. Here we show that this effect includes the release of the bulk of DNA from heterochromatic areas, in both human and mouse cells, without affecting the overall levels of heterochromatin associated histone H3 lysine 9 tri-methylation (3MK9H3). The release of DNA from the heterochromatic chromocenters in LANA-1 transfected mouse cells co-incides with the dispersion of the chromocenter associated methylcytosin binding protein 2 (MECP2). The localization of 3MK9H3 to the remnants of the chromocenters remains unaltered. Moreover, exogeneously expressed LANA-1 leads to the relocation of the chromocenters to the nuclear periphery, indicating extensive changes in the positioning of the chromosomal domains in the LANA-1 harboring interphase nucleus. Using a series of deletion mutants we have shown that the chromatin rearranging effects of LANA-1 require the presence of a short (57 amino acid) region that is located immediately upstream of the internal acidic repeats. This sequence lies within the previously mapped binding site to histone methyltransferase SUV39H1. We suggest that the highly concentrated LANA-1, anchored to the host genome in the nuclear foci of latently infected cells and replicated through each cell generation, may function as "epigenetic modifier". The induction of histone modification in adjacent host genes may lead to altered gene expression, thereby contributing to the viral oncogenesis.

## Background

Human herpesvirus virus 8 (HHV-8) is considered as the causative agent of Kaposi's sarcoma (KS) and is also associated with primary effusion lymphomas (PELs) and multicentric Castleman's disease (MCD). It is a gammaherpesvirus that shows sequence homology to Epstein-Barr virus (EBV) and herpesvirus saimiri (HVS) that are able to transform B (EBV) and T cells (HVS), respectively. Both viruses can cause malignant lymphomas [3]. HHV-8 encodes a large number of proteins that show structural similarities with cellular proteins involved in cellular proliferation, cell cycle regulation and immune modulation [4]. A human cyclin D homologue, vCYC, ORF72, a bcl-2 homologue, ORF16 [5], an IL-8-like G-protein coupled receptor, vGCRP, ORF74 [6] and interferon regulatory factors, vIRFs, ORFK9, ORFK10.5 [4] are among the genes that have been pirated by the virus.

The latency-associated nuclear antigen (LANA-1, LNA or LNA-1), encoded by ORF73, is one of few HHV-8 encoded proteins that is highly expressed in all latently infected tumor cells [7-9]. This suggests that LANA-1 plays a critical role in maintenance of latent HHV-8 infection. LANA-1 is a 222–234 kDa phosphoprotein with an acidic internal repeat domain flanked by a carboxy-terminal domain and an amino-terminal domain [9].

Constitutive expression of LANA-1 from its own promoter in transgenic mice induced splenic follicular hyperplasia due to an expansion of IgM+ IgD+ B cells and led to increased germinal center formation. LANA-1 expressing B-cell lesions could also progress to lymphomas [10].

LANA-1 acts as a transcriptional regulator. It has been shown to bind to p53 and to the retinoblastoma protein pRb. This leads to the inactivation of p53-dependent promoters and induction of E2F-dependent genes [11,12]. Together with the cellular oncogene H-ras, LANA-1 transforms primary rat embryo fibroblasts [13]. It can transactivate the promoter of the reverse transcriptase subunit of the human telomerase holoenzyme [14]. Activation of telomerase is a critical step in cellular transformation [15]. LANA-1 is also involved in transcriptional repression, however [16-18]. It can, moreover, interact with the mSin3/HDAC1 co-repressor complex [17]. It has been also shown to interact with and inhibit the ATF4/CREB2 transcription factor that interacts with the basic transcription machinery [19]. LANA-1 was also reported to bind two human chromosome-associated cellular proteins, MeCP2 and DEK [17].

RING3, a homology of the fsh (female sterile homeotic) gene product of *Drosophila*, interacts with LANA-1 [20]. This results in the phosphorylation of LANA-1. We have shown by immunofluorescence that LANA-1 can re-locate RING3 into heterochromatin regions and that LANA-1 and RING3 co-localize in the nuclear bodies of BCBL-1 cells. Exogenously expressed LANA-1 increased the expression of RING3 [2].

LANA-1 is associates with cellular chromatin and stays on the chromosomes during cell division [21]. It maintains the viral genomes during cell division by tethering the viral episomes to the chromosomes [22]. It binds directly to replication origin recognition complexes (ORCs) that are primarily associated with the terminal repeat (TR) region of the HHV-8 genome [23]. Binding of LANA-1 to TR confers transcriptional silencing, on the promoter of the neighbouring lytic gene K1 [24]. LANA-1 is believed to play an important role in the suppression of lytic viral genes and maintenance of viral latency. The key lytic regulator protein, RTA activates the expression of several lytic viral genes by interacting with recombination signal sequence-binding protein Jkappa (RBP-Jkappa), a transcriptional repressor and the target of the Notch signaling pathway. Importantly, LANA-1 also suppresses RTA activity by its direct binding to RBP-Jkappa [25].

Distinct regions of the N-terminus of LANA-1 are responsible for nuclear targeting and binding to human chromosomes [26]. The 1–22 N terminal residues of LANA-1 bind directly to an acidic patch on the core histone dimers H2A-H2B [27]. LANA-1 shows a characteristic cellular distribution. The HHV-8 episomes and the associated LANA-1 protein accumulate in irregularly shaped bodies in the interphase nucleus, preferentially at the border of heterochromatin [1]. It binds to human metaphase chromosomes in an apparently random fashion [21,26]. Exogeneously expressed C terminal part of LANA-1 preferentially concentrates to paired dots at pericentromeric and peri-telomeric regions of a subset of mitotic chromosomes [28]. A short 15 aa region in the C terminal part is responsible for the association with heterochromatin [29]. This chromatin-binding domain is required for multiple LANA-1 functions, such as the ability to bind to and replicate viral episomes, to modulate transcription, and to interact with the members of Brd chromatin binding proteins Brd2/RING3 and Brd4s [30-32].

We have previously shown that exogenous expression of LANA-1 induces a major re-organization of DNA staining patterns. Here we show that this reorganization leads to the disappearance of normal heterochromatin pattern in both human and mouse cells. In order to further characterize the effect of LANA-1 on heterochromatin we have compared its distribution in HHV-8 carrying cells and in non-infected but LANA-1 transfected cells in relation to different heterochromatin markers.

## Materials and methods

### Cell culture conditions and cell lines

The cells were cultured at 37°C at 5% CO_2 _in Iscove's modified Dulbecco's cell culture medium supplemented with 10% heat-inactivated fetal bovine serum (FBS), 100 U/ml penicillin and 100 U/ml streptomycin. The cells were passaged and split 1:5 every fourth day. The cell cultures were regularly tested for the absence of mycoplasma infection by Hoechst 33258 staining. The cell lines used in this study were the following: the HHV-8 infected human body cavity lymphomas BC-1 and BCBL-1; human breast carcinoma MCF-7; human osteosarcoma cell line Saos-2, human cervical carcinoma HeLa; immortalized mouse fibroblasts NIH3T3 and mouse fibrosarcoma L (American Type Culture Collection (ATCC).

### Transient transfection procedure

The human or mouse cells were grown on coverslips in six-well plates and transfected for 24–48 hours using a full-length LANA-1 cDNA inserted into a pcDNA1 vector or a LANA-1 deletion constructs [29]. An empty vector or a pBabe EBNA-5 construct created by us was used for control transfections. Transfection of cells was made according to the manufacturer's instructions using FuGene6 (Roche).

### Immunofluorescence microscopy

The transfected cells (grown on coverslips) or body cavity lymphoma cell lines (cytospinned on to glass slides) were fixed in methanol: acetone (1:1) at -20°C for 20 min. The re-hydration of cells was done in PBS for 20 min at room temperature. The following antibodies were used in this study for immunofluorescence straining: human anti-LANA KS2 (antiserum, a gift from Attila Juhasz, the Dermatology Unit of Debrecen Medical School, Hungary), rabbit polyclonal anti-tri-methyl K9 histone H3 (A gift from Dr Prim Sing); rabbit polyclonal IgG anti-mouse MeCP2 (reacting with both human and murin MeCP2) (Upstate); FITC-conjugated swine anti-rabbit (DAKO); rhodamine-conjugated rabbit anti-human (DAKO); FITC-conjugated rabbit anti-human (DAKO) or Texas red-conjugated horse anti-mouse (Vector) were used as secondary antibodies. The different combinations of primary and secondary antibodies are specified in respective figures. The control transfection of pBabe-EBNA-5 was stained with a mouse monoclonal anti-EBNA-5 (JF186)[33]. Texas red-conjugated horse anti-mouse (Vector) was used as secondary antibody. The antibodies were diluted in blocking buffer (2% BSA, 0.2% Tween-20, 10% glycerol, 0.05% NaN_3 _in PBS). The primary antibodies were incubated in a humid chamber at room temperature for one hour followed by three washes with PBS. Incubation with the secondary antibodies was done for one hour in humid chamber at room temperature. Double staining between LANA-1 and the different chromatin-associated proteins were done as follows: rabbit anti-tri-methyl K9 H3 or rabbit anti-MeCP2, FITC-conjugated swine anti-rabbit, normal rabbit, human anti LANA-1 and at last rhodamine conjugated rabbit anti-human. DEK double staining: mouse anti-DEK, Texas red conjugated horse anti-mouse, normal mouse, human anti-LANA1 and FITC conjugated rabbit anti-human. The DNA was stained with Hoechst 33258. Each incubation step was followed by three washes in PBS.

The images were captured with one of the following systems: Leitz DM RB wide field fluorescence microscope equipped with a Hamamatsu dual mode cooled CCD camera C4880 where the images were recorded and analysed on a Pentium PC computer equipped with an AFG VISION*plus*-AT frame grabber board using Hipic 4.0.4 (Hamamatsu), Image-Pro Plus (Media Cybergenetics). Digital images were assembled using Adobe PHOTOSHOP software. Alternatively a Zeiss Axiophot microscope was used to reconstitute images from a series of optical sections that were de-blurred by removing the out-of-focus blur using a nearest neighbor de-convolution algorithm developed by us. On this system the images were captured with a PXL cooled camera (Photometrics, Munich, Germany) and analyzed using our own image capture and analysis programs developed on ISee graphical programming language running under Mandrake LINUX OS on a Pentium PC computer [34]. Confocal images and very large field mosaics were captured using our custom built dual mode Ultraview (combined RS and LCI) system (Perkin Elmer) using imaging automations QuantCapture 4.0 and QuantCount 3 that we have developed using OpenLab Automator programming environment (Improvision). 3D reconstitution was carried out using Volocity (Improvision) or ImageJ programs.

## Results

### Effect of exogeneously expressed LANA-1 on nuclear structures of human cells

**Figure 1:**
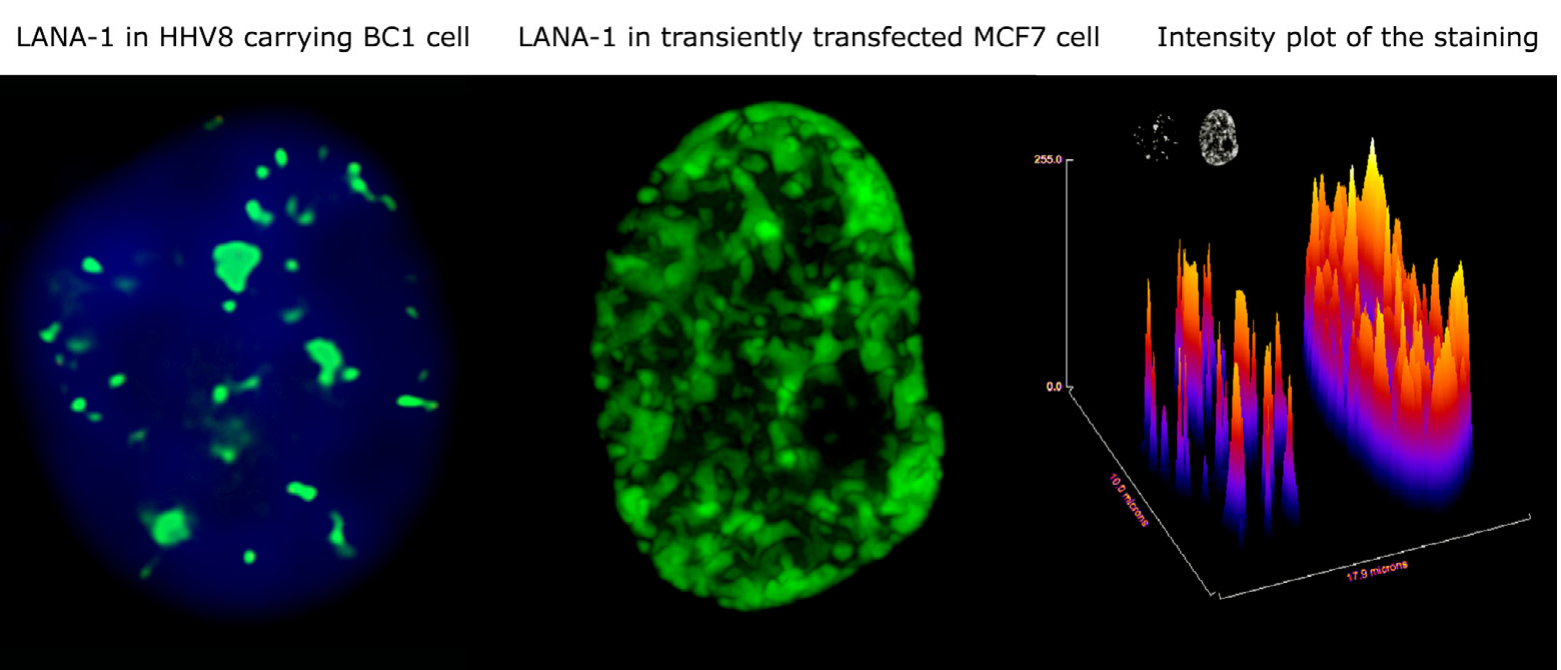
Comparison of expression levels of virus encoded and exogeneously introduced LANA-1 in the nucleus of HHV-8 positive BCBL-1 body cavity lymphoma (left) and HHV-8 negative MCF7 breast carcinoma cell (middle). Immunofluorescence staining (green) using human anti-LANA-1 serum detected by FITC conjugated mouse anti-human immunoglobulins. The images are Z axis projections of stacks of 10 optical sections, 0.5 micrometer apart, captured from identically stained and processed nuclei using an automated wide field fluorescence microscope. The counterstaining of BCBL-1 DNA with Hoechst 33258 (blue) is shown for easier orientation. The 3D projection of the plot of the measured staining intensity (right) illustrates that the nuclear foci of latently infected cells contain comparable amount of LANA-1 to the transiently transfected ones.

**Figure 2:**
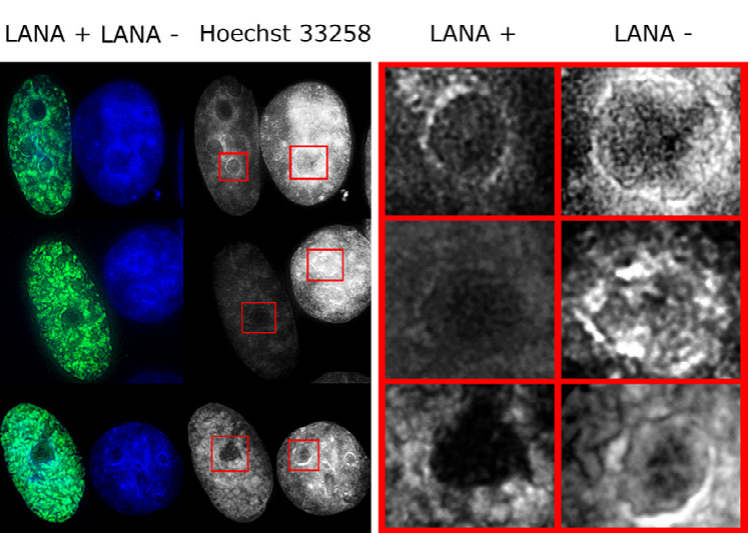
LANA-1 (green) dissolves DNA (blue) from perinucleolar heterochromatin in transfected MCF7 cells. Increasing amount of transfected LANA-1 (compare top to the middle or bottom panels) leads to the elimination of the DNA staining (blue) in the perinucleolar heterochromatic rings. Adjacent non-transfected cells serve as controls. Right panel shows magnified pictures of chromatin organization of the selected nuclear areas selected by red border boxes in the left panel. The middle and bottom panels represent the two distinct types of chromatin effects: The smoothing out of the chromatin staining (middle panel) versus induction of newly condensed chromatin cords (bottom panel). Importantly both changes lead to the elimination of perinucleolar heterochromatin.

**Figure 3:**
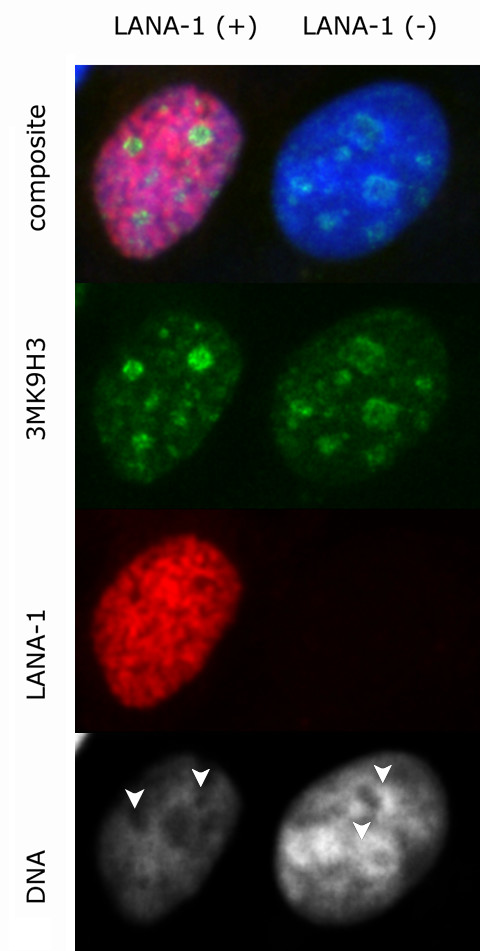
Dissolution of DNA from perinucleolar heterochromatin is not accompanied by the release of trimethylated lysine 9 histone H3 (3MK9H3) – green immunofluorescence staining in LANA-1 transfected cells (red). 3MK9H3 staining clearly identifies perinucleolar areas (white arrows) with diminished heterochromatic DNA staining in the transfected cells.

**Figure 4:**
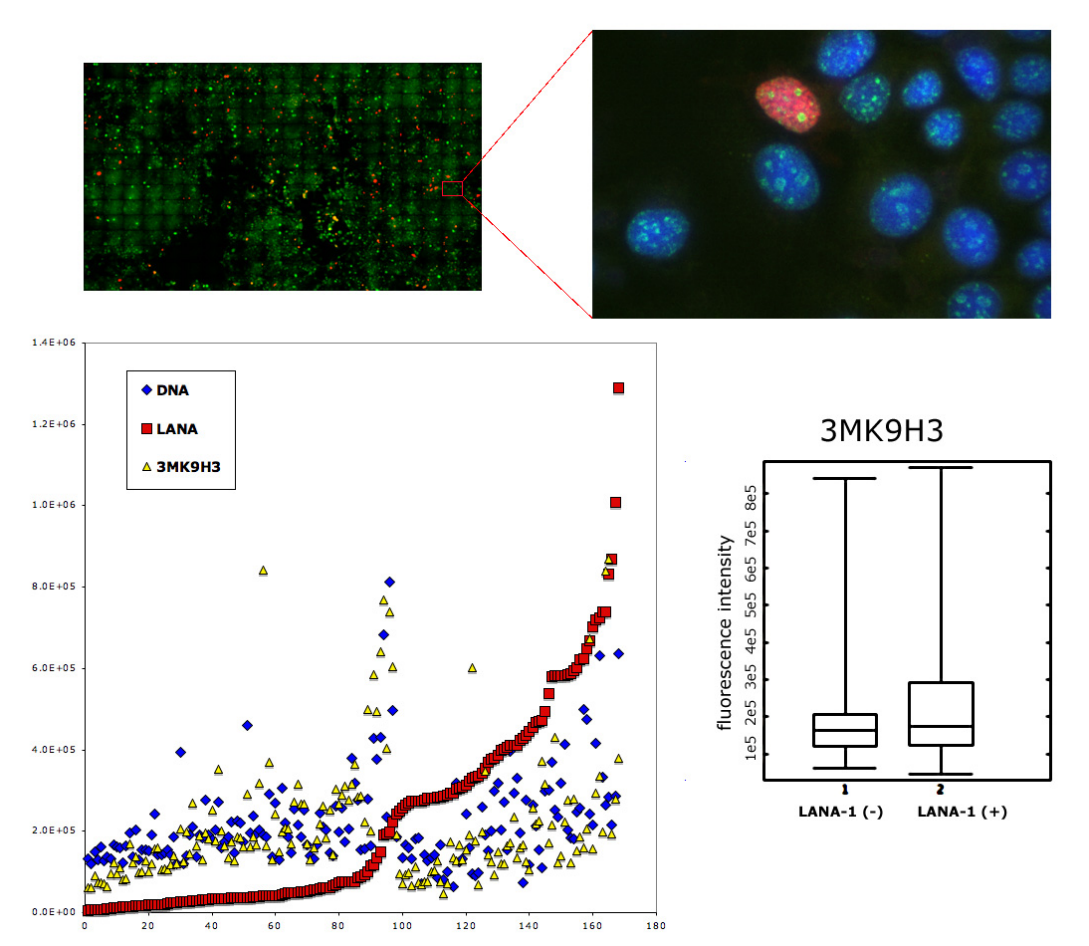
LANA-1 expression does not effect 3MK9H3 (green) levels as measured using extended field laser scanning microscopy (EFLCM) that automatically captured 300 adjacent fields as a mosaic of Z projected images of 12 optical sections each. The total fluorescence intensity measurement for 3MK9H3, DNA (blue) and LANA-1 (red) of the individual nuclei is plotted in the order of increasing amount of LANA-1. The amount of 3MK9H3 staining is also compared on population levels of LANA-1 positive and negative nuclei on a box chart.

HHV-8 infected cells harbor LANA-1 in a spatially strictly restricted manner. The majority of LANA-1 is associated with well-defined nuclear areas that also contain viral episomes (here referred as LANA bodies). Although there are multiple binding sites in the viral terminal repeat, we have estimated that the amount of LANA-1 concentrated in the nuclear foci is orders of magnitude higher than the one that can form direct contact with the viral DNA. In order to model the effect of high LANA-1 concentration on the organization of chromatin in the neighborhood of LANA bodies, we overexpressed LANA-1 in transiently transfected MCF7, HeLA or Saos-2 cells. The level of expression was determined by measuring the fluorescence signal intensities on identically processed, immunostained slides, using manual, semi-automated or fully automated wide-field or spinning disc confocal fluorescence microscopy. The measurement data demonstrated that the focal expression levels of LANA-1 in the LANA bodies were comparable to the levels reached in the transiently transfected cells (Figure 1). Expression of LANA-1 in comparable quantities that occur in the LANA bodies has led to profound rearrangement of nuclear structures in the transiently transfected cells. In human cells this is most prominently demonstrated by the effect on perinucleolar heterochromatin. Two major, distinct type of chromatin alterations were observable. In a fraction of transfected cells LANA-1 overexpression led to the almost homogeneous elimination of chromatin staining pattern (Figure 2 middle panel) whereas in other cells a novel condensed chromatin pattern appeared, that was somewhat similar to the morphology of premature chromosome condensation observable in mitosis/interphase cell hybrids (Figure 2 bottom panel). Importantly both type of chromatin change had a major effect on the heterochromatin. Increasing levels of LANA-1 led to the disappearance of heterochromatin from the perinucleolar areas as defined by Hoechst 33258 staining (compare the top panel of Figure 2 to the middle or bottom panel). Importantly this effect was not associated by a similar release of the heterochromatin marker trimethylated lysine 9 on histone H3 (3MK9H3) from the perinucleolar areas. On the contrary 3MK9H3 positive heterochromatin remnants appeared to be collapsed into smaller spherical structures (Figure 3). Measuring the amount of 3MK9H3 in the nuclei of MCF7 cells, 48 hours after transfection, using automated Extended Field Laser Confocal Microscopy (EFLCM), we found no significant difference between the total amount of 3MK9H3 in the untransfected and LANA-1 transfected cells (Figure 4).

### Effect of exogenously expressed LANA-1 on nuclear structures of mouse cells

**Figure 5:**
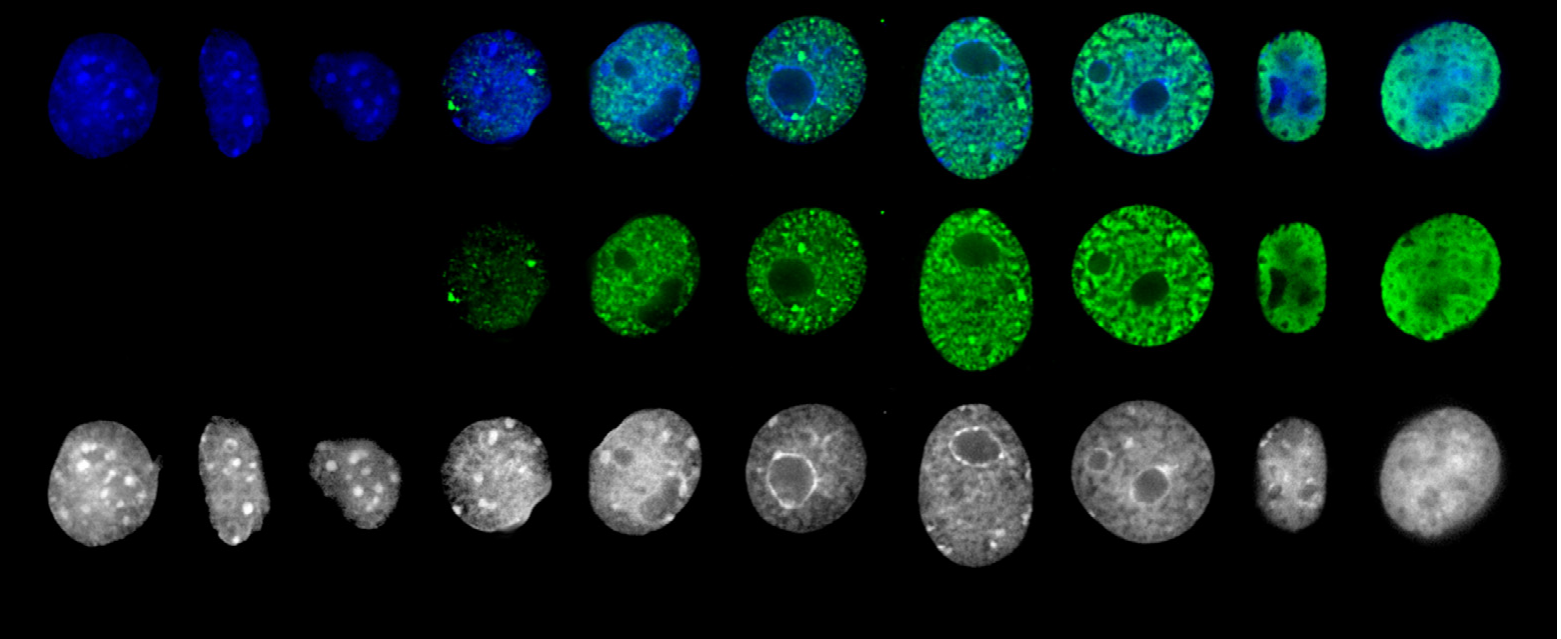
Effect of LANA-1 on mouse pericentromeric heterochromatin organized as chromocenters. Increasing amount of LANA-1 (green) leads to gradual disappearance of chromocenters in the transfected nuclei of murine L-cells. DNA stained with Hoechst 33258 (blue).

**Figure 6:**
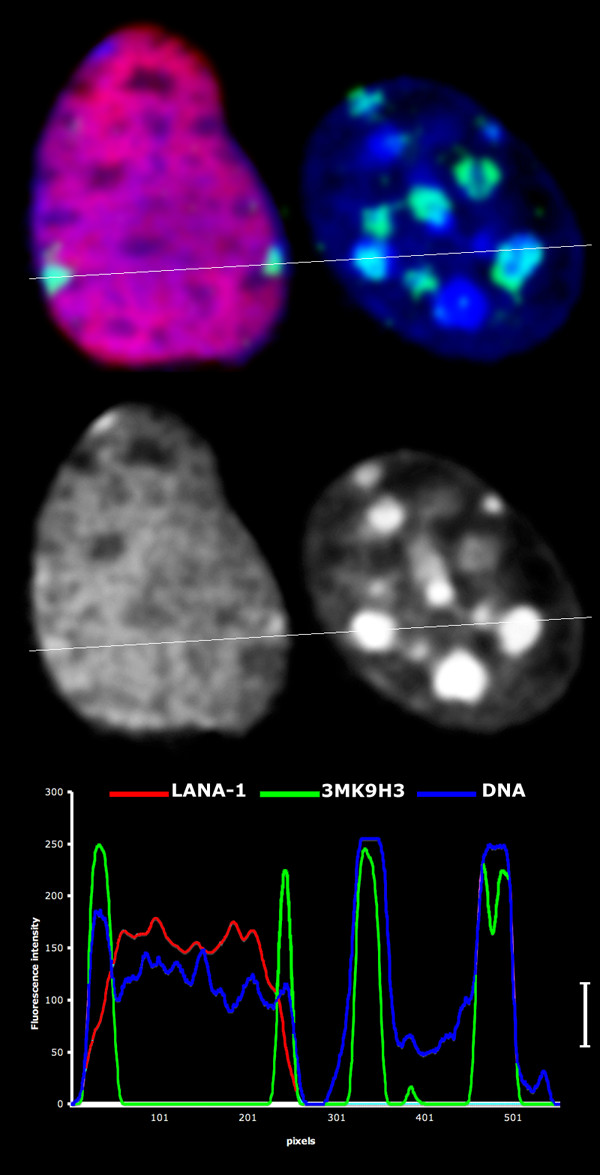
High resolution comparison of LANA-1 (red) positive L-cell nucleus with an adjacent non-transfected cell in a single optical section that slices both nuclei in the middle level. The intensity plot is recorded along the white line and demonstrate a massive release of the bulk DNA (blue) from the chromocenters in the transfected cell without effecting the 3MK9H3 (green) levels in the remnants of the chromocenters (white staple on the right side of the line plot).

**Figure 7:**
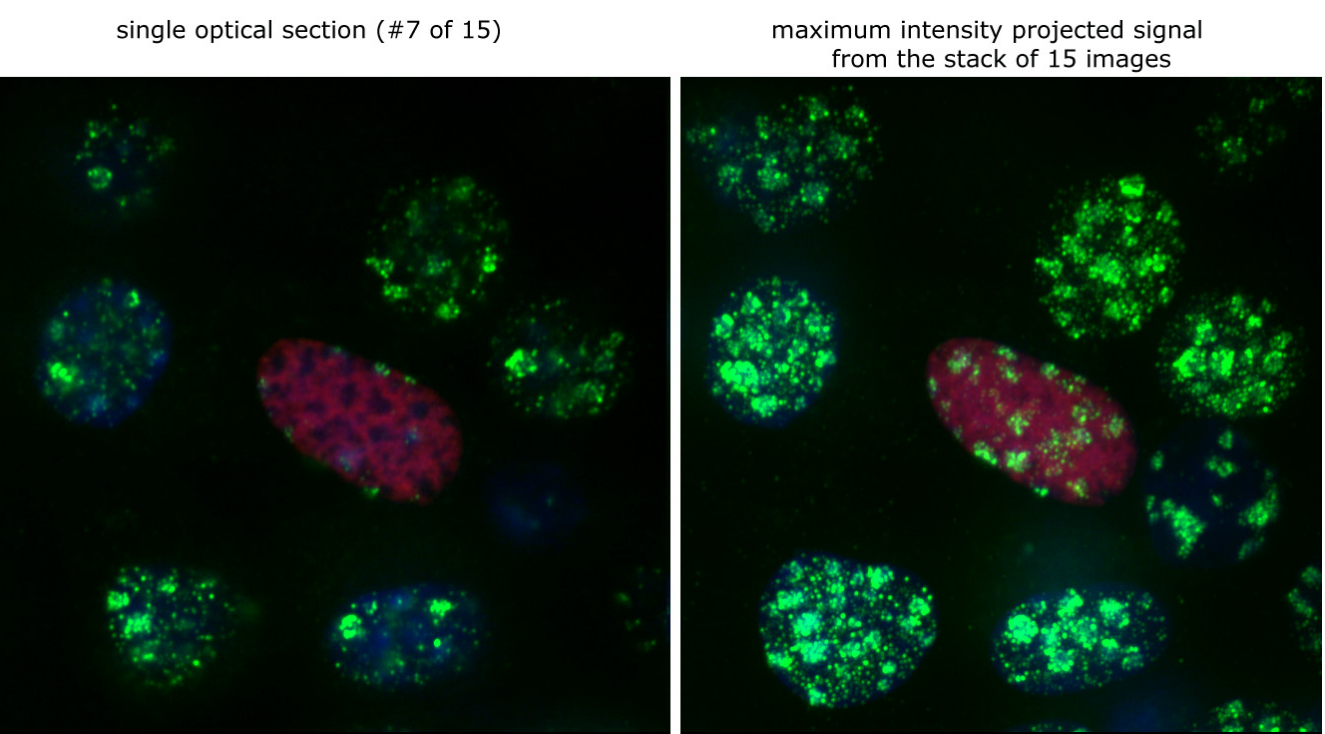
Although single optical sections (left) of LANA-1-transfected nuclei may suggest extensive decrease in 3MK9H3 staining, reconstitution of the summarized staining signal from the entire stack of 15 images (right) shows no detectable alteration in the total levels of 3MK9H3 in mouse L cell nuclei. (LANA-1 – red, 3MK9H3 – green, DNA – blue).

**Figure 8:**
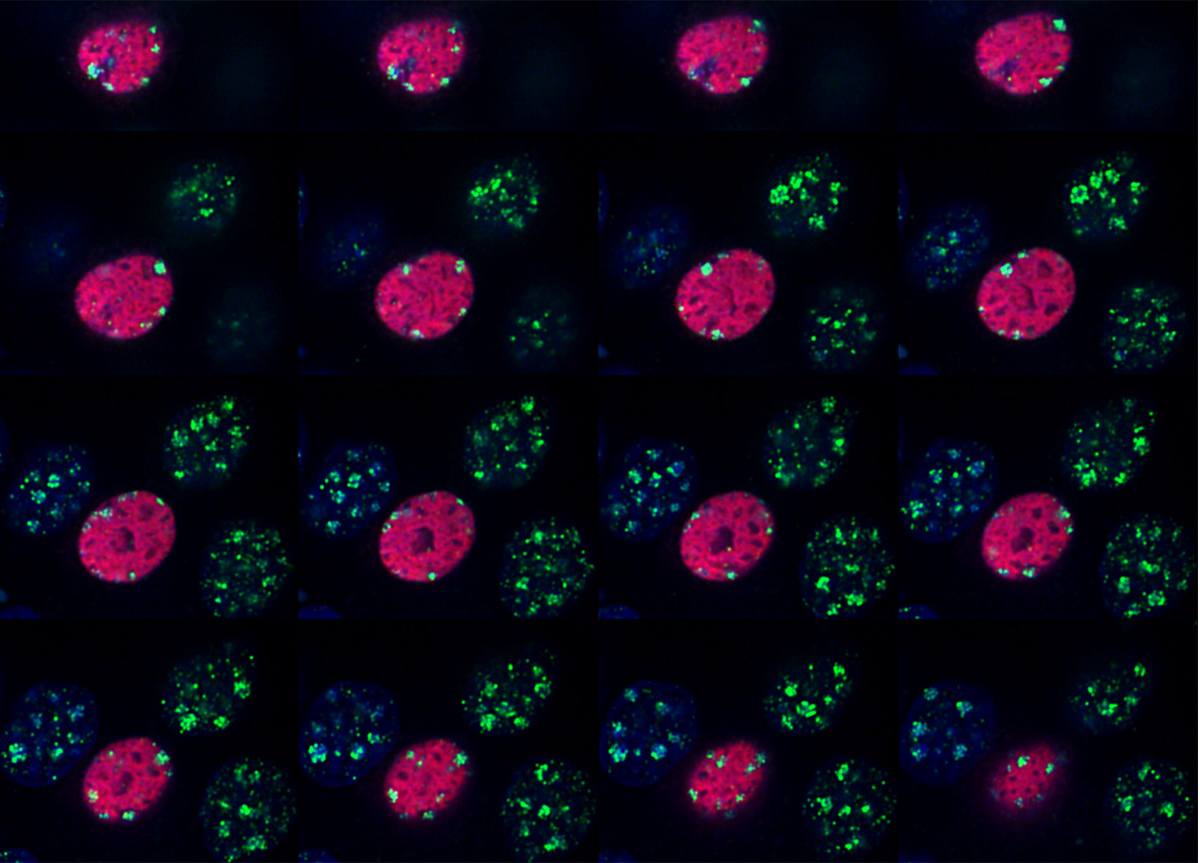
Careful examination of the series of sixteen individual optical sections along the Z axis reveals that LANA-1 induces a major rearrangement in the positioning of 3MK9H3 positive chromocenter remnants from the inside of the nucleus to the periphery of the nucleus. (LANA-1 – red, 3MK9H3 – green, DNA – blue).

**Figure 9:**
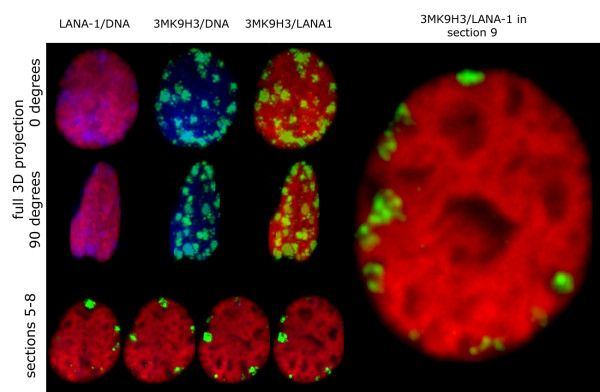
3D reconstitution of the triple-stained nuclei shows that LANA-1 staining and the 3MK9H3 positive remnants of the heterochromatic chromocenters are non-overlapping. See the full rotational image sequences in the supplementary material [see Additional file 1]. The selected middle sections (5–9) demonstrate the extensive peripheral localization of the chromocenters. High magnification representation of a single section shows mutual avoidance of 3MK9H3 staining and LANA-1 distribution. 3D reconstitution of LANA-1 transfected nuclei also reveals well-defined nuclear areas that are devoid of LANA-1 staining. The identity and molecular content of these areas are currently unknown but they apparently include the nucleoli and the chromocentric remnants. The 3 dimensional digital cast of the LANA-1 negative areas in relation to 3mK9H3 and DNA staining is shown as image sequence in the supplementary material [see Additional file 2]. (LANA-1 – red, 3MK9H3 – green, DNA – blue).

**Figure 10:**
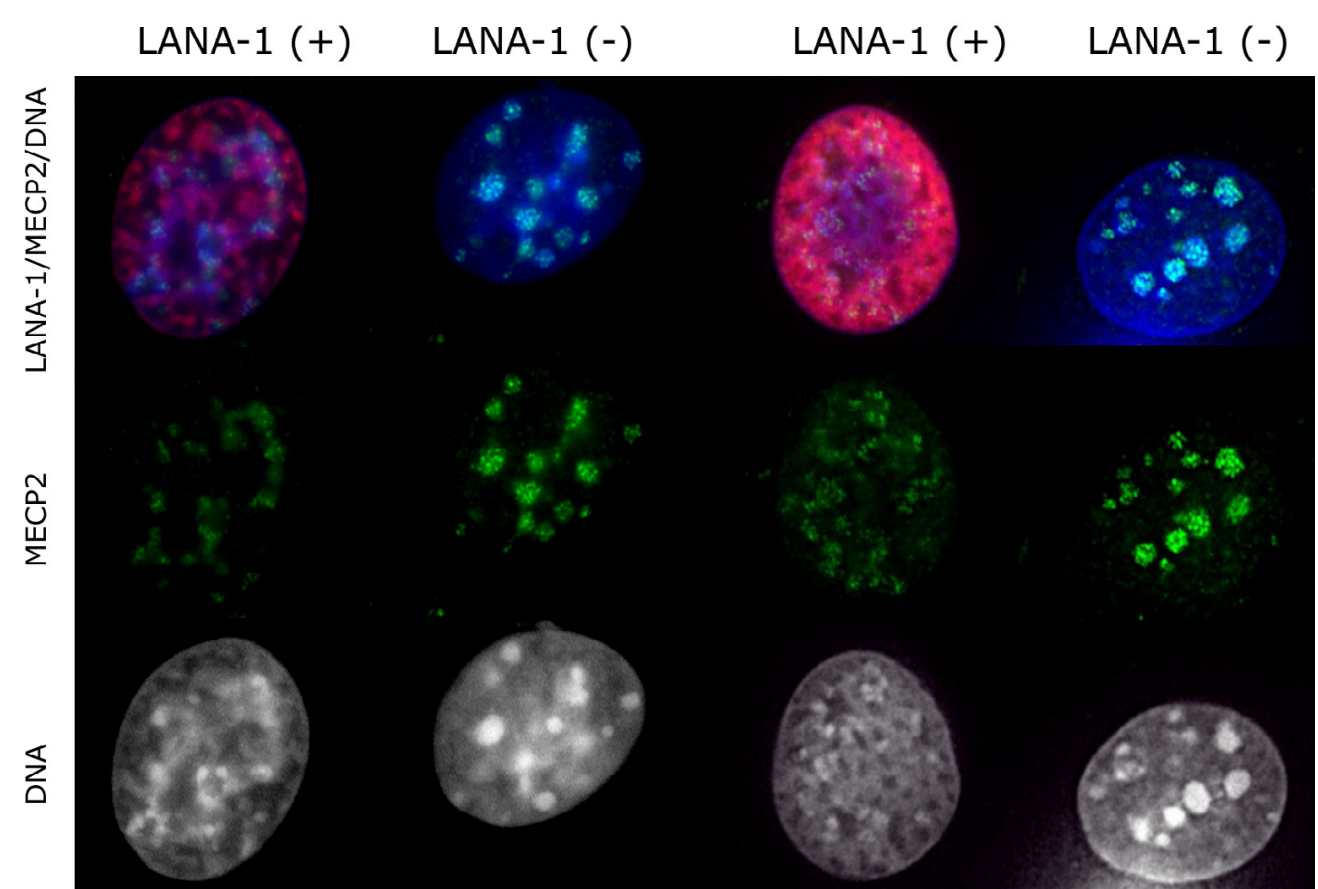
Unlike 3MK9H3, the heterochromatin associated methyl cytosine binding protein 2 (MECP2) shows an extensive release from the chromocenters of LANA-1 transfected L cells both at moderate (left) and high expression levels (right). (LANA-1 – red, MECP2 – green, DNA – blue).

**Figure 11:**
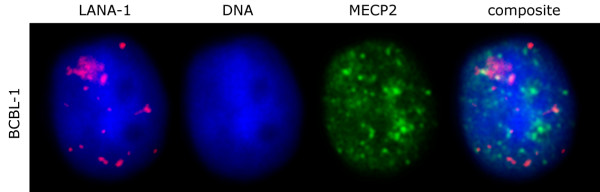
Importantly however MECP2 is absent from the LANA bodies in BCBL-1 cells that harbor latent HHV-8 genomes. (LANA-1 – red, MECP2 – green, DNA – blue).

Morphological analysis of heterochromatic structures is cumbersome in human cells because of the rather diffuse border between the euchromatin and heterochromatin areas. The discrimination between the two types of chromatin is much easier in mouse cells where the pericentromeric alfa-satellite repeats are organized in very well defined heterochromatic chromocenters. We have previously shown that LANA-1 has retained its ability to target mouse heterochromatin in BCBL-1/Sp2 human/mouse synkaryon hybrids [1]. In order to test the effect of LANA-1 on mouse chromocenters we have transfected A9 and L cells as well as NIH3T3 fibroblasts with LANA-1. All tree lines showed the same effect. Increasing amount of LANA-1 has led to the disappearance of chromocenters by Hoechst 33258 staining and formation of condensed chromatin at the nuclear periphery or in the perinucleolar areas (Figure 5). Interestingly the disappearance of the bulk of the DNA from the chromocenters, as illustrated on single narrow confocal sections of transfected and control nuclei (Figure 6) was not followed by the disappearance of 3MK9H3 staining (Figure 6). As in human cells, LANA-1 transfected mouse cells contained similar amounts of 3MK9H3 as non-transfected cells and both the number and the staining intensity of individual 3MK9H3 foci was unaltered. Importantly, however, the localization of 3MK9H3 foci was dramatically changed (Figure 7). Whereas in the untransfected cells the 3MK9H3 foci were evenly distributed throughout the entire nucleus, they were almost exclusively localized to the nuclear periphery in the transfected cells (Figure 8). High resolution optical sectioning and 3D reconstitution of the confocal image series showed that the relocation of the foci was an early event that has preceded the release of Hoechst stained chromatin from the chromocenters (Figure 9) [see Additional files 1 and 2]. The release of Hoechst stained DNA from the chromocenters was accompanied with a major rearrangement in the staining pattern of an other heterochromatin binding factor, the methyl cytosine binding protein 2 (MECP2) in mouse L-cells. Increasing expression of LANA-1 led to the gradual dissolution of MECP2 foci that were stringently associated with the chromocenters in untransfected cells. In transfected cells, MECP2 was released from the foci and appeared in the areas of the newly formed condensed chromatin bundles but showed no co-localization with LANA-1 itself (Figure 10). This lack of co-localization was also consistent with the absence of co-localization between LANA-1 and MECP2 in HHV-8 carrying BCBL-1 cells (Figure 11).

### Mapping the LANA-1 region required for the chromatin effects using deletion mutants

**Figure 12:**
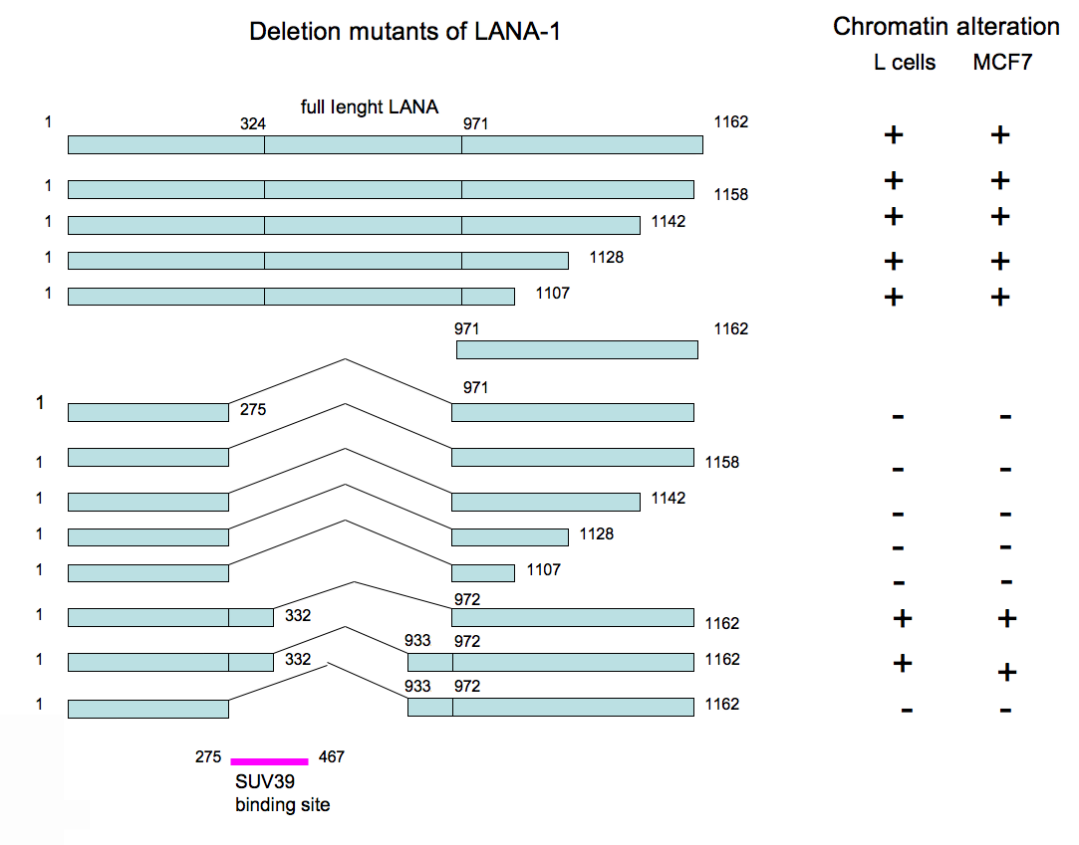
Series of deletion mutants affecting both the C terminus and the central acidic repeats identify the N terminal area immediately preceding the central acidic repeats as an area that is required for the chromatin modifying effects. This area closely overlaps with the previously identified site of histone methyl transferase SUV39H1.

**Figure 13:**
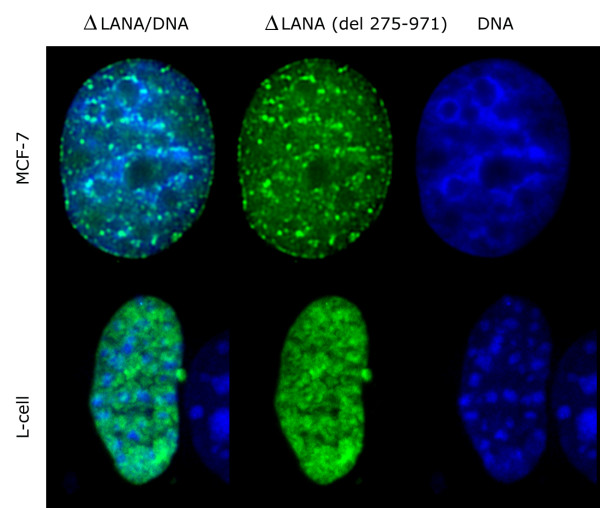
Deletion mutant (del 275–972) lacking the central acidic repeats and the adjacent N terminal region show effective targeting to the surface of heterochromatin both in human MCF7 and in mouse L-cells without any visible effect on the chromatin organization or positioning of chromocenters. (ΔLANA-1 – green, DNA – blue).

**Figure 14:**
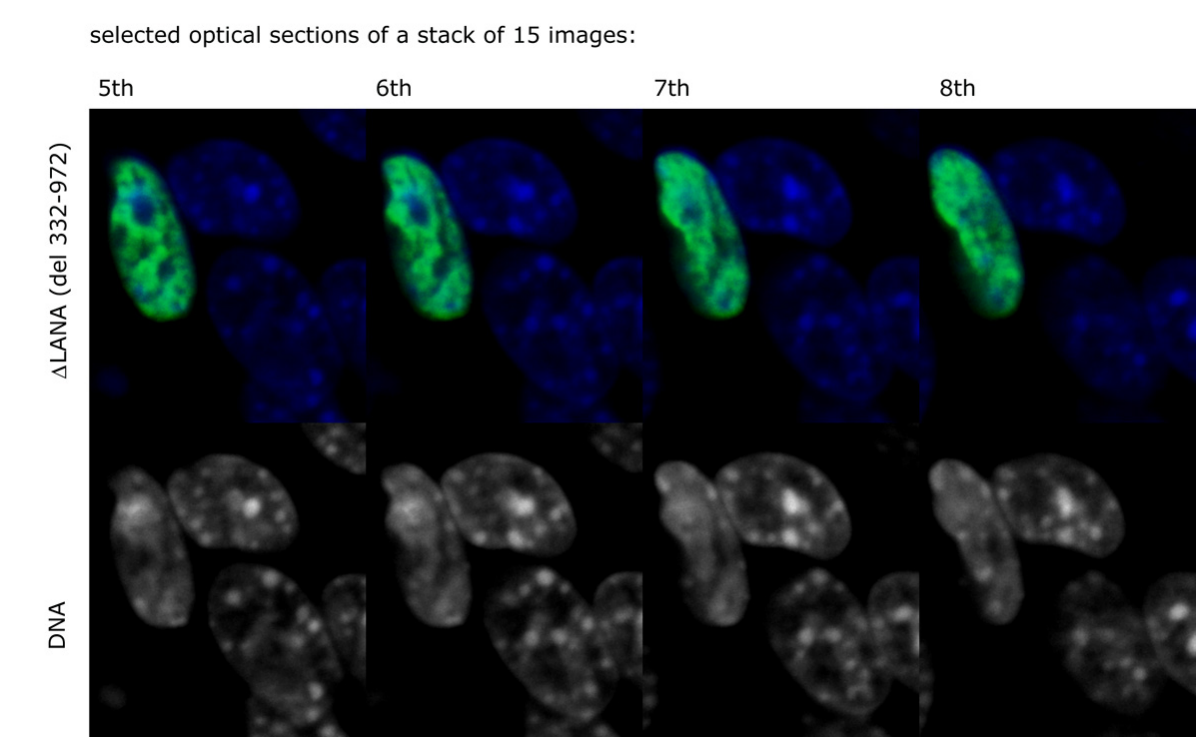
Chromatin dissolution effect is preserved in the deletion mutant LANA (ΔLANA-1 – green, DNA – blue). (del 332–972) as shown in four adjacent confocal sections of transfected L cell nuclei.

We have tested a series of C-terminal deletion mutants that did or did not contain the central acidic repeat regions for their effect on the chromatin organization in human and mouse cells. The C terminal truncation had no effect on LANA-1 induced chromatin rearrangement, while deletions of the central acidic repeat eliminated this effect (summarized in Figure 12). Deletion mutants lacking the central repeats (delta mutants) retained the capacity to target the surface of heterochromatin both in human and mouse cells, but had no effect on the organization of chromatin itself (Figure 13). To identify the region that was involved in the chromatin reorganizing of LANA-1 more precisely, we tested mutants that retained the immediate neighboring sequences of the central repeats. We found that a mutant (del 332–972) that lacked the internal repeats but retained the immediate upstream region that precedes the DE repeats was active in rearranging the chromatin (Figure 14). The compiled data showed that this 57 amino acids long region (aa. 275–332) was required for the chromatin effects. Importantly this area is overlapping with the previously mapped binding site for the histone methyl transferase SUV39H1 [35].

## Discussion

HHV8 carrying cells can harbor up to a few dozen viral episomes that localize in discrete nuclear compartments delineated by LANA-1. LANA-1 as multifunctional virally encoded protein, involved in the maintenance of the viral episomes, regulation of viral latency, transcriptional regulation of viral and cellular genes and impairment of cell cycle checkpoints [36]. Several of these functions may play a role in the HHV8 induced malignant transformation of Kaposi sarcoma and body cavity lymphoma cells. In this paper we have presented additional evidence that LANA-1 may also have fundamental effects on the organization of the interphase nucleus. They are most clearly seen in mouse cell nuclei where the pericentromeric heterochromatin forms easily recognizable chromocenters. The bulk of the heterochromatin associated DNA is released from the chromocenters without affecting their 3MK9H3 content. This suggests indirectly, that a large part of the DNA that is associated with the chromocenters contains nucleosomes that are not trimethylated on the 9^th ^lysine of histone H3, a modification that is considered to be the hallmark of heterochromatin organization. Our data suggest that chromatin with 3MK9H3 modified nucleosomes is interspersed with nonmodified nucleosomes inside the heterochromatic areas and that LANA-1 has a strong dispersing effect on this type of chromatin. It is likely that this chromatin fraction is heavily methylated on CpG sites, indicated by the profound effect on the distribution of MECP2 protein. The release of MECP2 from heterochromatin is probably not due to a direct stable interaction with LANA-1 because the two proteins showed no co-localization in either the transfected or in the virus carrying cells. LANA-1 binds MECP2 in vitro and in co-immunoprecipitation experiments. The presented morphological distribution data suggest that this interaction in vivo may occur outside of the LANA bodies in the virus infected cells and the complex may have a short half life.

3MK9H3 labelled chromocenter remnants may also function as markers of the centromeric side of interphase chromosome domains in mouse cells. The relocation of these foci from the interior of the nucleus to the nuclear periphery indicates major reshuffling of individual chromosomes in the LANA-1 containing interphase nuclei. The mechanism that determines the position of the different chromosomal domains in the nucleus is debated. Several theories were proposed to explain the relative localization and movements of chromosomal domains within the interphase nucleus. They can be referred to as the gene density theory, chromosome size theory and the mitotic pre-set theory (position within the metaphase plate), as reviewed by [37,38]. The relative position and mobility of individual chromosomes have an obvious bearing on the occurence of chromosomal translocations. Our findings raise the possibility that LANA-1 may contribute to the development of the numerous chromosome rearrangements and translocations, seen in HHV8 carrying body cavity lymphoma lines [21].

The analysis of deletion mutants has revealed an area, corresponding to the SUV39H1 binding site, is required for the LANA-1 induced chromatin effects. SUV39H histone methyltransferases govern histone H3 lysine 9 (H3–K9) methylation at the pericentric heterochromatin and induces a specialized histone methylation pattern that differs from the broad H3–K9 methylation present at other chromosomal regions. Trimethyl H3K9 is a marker of constitutive heterochromatin. Mouse chromocenters are clusters of late-replicating pericentric heterochromatin that contains heterochromatin associated protein 1 (HP1) bound to 3M-H3K9. It has been suggested that H3–K9 methylation may function as a major "switch" for the functional organization of chromosomal subdomains. The viability of SUV39H-deficient mice is severely impaired suggesting that the SUV39H HMTases are important epigenetic regulators for mammalian development [39]. The chromosomal instability of SUV39H-deficient mice is associated with an increased tumor risk and perturbed chromosome interactions during male meiosis. Mouse cells, deficient for both SUV39H1 and H2 show very similar Hoechst 33258 staining patterns to the ones that we have described in the present paper. Pericentromeric H3–K9 tri-methylation is believed to play an important role in protecting genome stability. Our data suggest that LANA-1 does not alter the overall level of H3–K9 tri-methylation but releases DNA from heterochromatic areas. Considering that the viral episomes are anchored to the heterochromatin borderlines, the virus may need a molecular mechanism to prevent overall heterochromatization, as seen in the positional effect variegation described in Drosophila and regulated by the fly variant of SUV39 (Su(var)3–9 Suppressor of variegation 3–9). LANA-1 may play a crucial role in the regulation of histone modifications of the viral genes and those cellular genes that are in close proximity to the viral episome. Indeed, LANA-1 can mediate the inactivation of lytic cycle associated genes in the neighborhood of the terminal repeats through local interaction with SUV39H1. More generally, LANA-1 may have a complex effect on the epigenetic modifications of the viral and host genome. It interacts with a set of proteins that influence histone modifications responsible for heterochromatin formation. Its interaction with retinoblastoma protein (RB1) may also have a direct effect on heterochromatin formation. Gonzalo et al. (2005) showed that members of the RB family are directly involved of in heterochromatin formation, through their interaction with HMTs, independently of the interaction with E2F. LANA-1 also binds to the de novo DNA methyltransferases DNMT3A [40] that in turn bind to another euchromatic histone methyltransferase SETDB1/ESET. The latter can also trimethylate H3–K9 [41].

In the view of the massive effect of exogenously expressed LANA-1 on chromatin structure in transfected cells it is reasonable to assume that endogenous LANA in virus carrying cells has a similar effect on the neighboring chromatin of LANA bodies that contain comparable quantities of LANA in well circumscribed areas.

The focal concentration of LANA-1 in latently infected cells with fixed anchorage to certain chromosomal sites raises the possibility for an a new paradigm of the *"epigenetic modifier" *that may generate locally altered chromatin states in regions of the host genome, affected at random. Once established it may be propagated over series of cell divisions. The altered epigenetic state may affect target genes involved in growth control. Through this mechanism the anchored LANA bodies may directly participate in the virus induced cellular transformation. This scenario implies the experimentally testable prediction that the altered expression of some cellular genes at the LANA body anchorage sites may contribute to the generation of HHV-8 harboring tumors such as Kaposi sarcomas and body cavity lymphomas. Systematic cloning effort using chromatin immunoprecipitation (Chip) material should enrich for these sequences. To identify the important target sequences, individual clones from one anti-LANA Chip cloning experiment should be arrayed. (Obviously clones with viral DNA inserts should be excluded). This array should be repeatedly probed with labeled Chip material from a number of HHV-8 bearing tumors from different individuals (Chip-on-chip). Clones that consistently light up with probes from a number of different tumors may be good candidate targets for the *"epigenetic modifier" *effect of the anchored viral episome.

## Supplementary Material

Additional file 1 Full rotational image sequences of the 3D reconstitution of the triple-stained nuclei in LANA-transfected L cells. LANA-1 staining and the 3MK9H3 positive remnants of the heterochromatic chromocenters are non-overlapping. (LANA-1 – red, MECP2 – green, DNA – blue). Click here for file

Additional file 2 Movie showing the LANA negative areas in the nucleus of L cells transfected with LANA. Full rotational image sequences of the 3 dimensional digital cast of the LANA-1 negative areas in relation to 3mK9H3 and DNA staining. (LANA-1 – red, MECP2 – green, DNA – blue). Click here for file
